# National survey of evaluation practices and performance-guided resource allocation at German medical schools

**DOI:** 10.3205/000270

**Published:** 2019-04-18

**Authors:** Sarah Schiekirka-Schwake, Janina Barth, Josef Pfeilschifter, Reinhard Hickel, Tobias Raupach, Christoph Herrmann-Lingen

**Affiliations:** 1 Studiendekanat, Universitätsmedizin Göttingen, Germany; 2 Präsidium, Medizinischer Fakultätentag, Berlin, Germany; 3 Dekanat des Fachbereichs Medizin, Goethe-Universität Frankfurt, Germany; 4 Dekanat der Medizinischen Fakultät, Ludwig-Maximilians-Universität München, Germany; 5 Klinik für Kardiologie und Pneumologie, Universitätsmedizin Göttingen, Germany; 6 Health Behaviour Research Centre, University College London, United Kingdom; 7 Klinik für Psychosomatische Medizin und Psychotherapie, Universitätsmedizin Göttingen, Germany; 8 Arbeitsgemeinschaft der Wissenschaftlichen Medizinischen Fachgesellschaften e.V., Berlin, Germany

**Keywords:** evaluation, evaluation practice, performance-based funding allocation

## Abstract

**Background:** Little is known about evaluation practices as well as performance-oriented allocation of resources according to teaching quality at German medical schools. For this reason, the Association of the Scientific Medical Societies in Germany and the German Association of Medical Faculties aimed to analyse current practices at German medical schools.

**Methods:** Data were collected by a questionnaire which was sent to all medical schools in Germany.

**Results: **30 medical schools with 33 undergraduate medical programs participated in the survey (response rate: 83%). The evaluation tools used at these schools mainly assessed structural and procedural aspects of teaching and were designed to obtain overall student ratings of teaching quality. Evaluation tools were quite heterogeneous across the sample, and some uncertainty remained with regard to the psychometric properties of these tools and whether they meet international quality standards. Various algorithms underlying resource allocation for teaching are being used, but most focus on quantity rather than quality of teaching.

**Conclusion:** A nationwide agreement on a generalizable definition of high-quality teaching is desirable. At the same time, reliable and valid tools measuring teaching quality need to be identified and/or created. This could be accomplished through a wider collaboration of medical schools and could represent an advancement for the allocation of resources for high-quality teaching.

## Zusammenfassung

**Hintergrund:** Bislang fehlen umfassende Daten zu Evaluationspraktiken und Leistungsorientierter Mittelvergabe (LOM) in der Lehre für die deutschen medizinischen Fakultäten. Vor diesem Hintergrund haben sich die Arbeitsgemeinschaft der Wissenschaftlichen Medizinischen Fachgesellschaften e.V. (AWMF) und der Medizinische Fakultätentag (MFT) das gemeinsame Ziel gesetzt, die Praxis der Evaluationen und LOM in der Lehre (LOM-Lehre) an den medizinischen Fakultäten in Deutschland zu analysieren.

**Methoden:** Die Datenerhebung erfolgte mittels Fragebogen, der an alle medizinischen Fakultäten in Deutschland gesandt wurde.

**Ergebnisse:** An der Befragung nahmen 30 Fakultäten mit insgesamt 33 Studiengängen teil (Rücklauf: 83%). Die an den Fakultäten eingesetzten Erhebungsinstrumente erfassen vorrangig strukturelle und prozedurale Aspekte sowie einen Gesamteindruck der Lehre. Zwischen den Fakultäten herrscht bezüglich der verwendeten Instrumente eine recht hohe Heterogenität. Teilweise bleibt unklar, inwiefern die Erhebungsinstrumente internationalen Qualitätsstandards genügen. Die finanzielle Honorierung der Lehre erfolgt überwiegend im Rahmen der Grundausstattung bzw. nach Kriterien der Lehr-Quantität. Qualitätsbasierte Mittelzuweisung spielt eine eher untergeordnete Rolle.

**Schlussfolgerung**: Eine möglichst bundesweite Konsentierung eines Leitbilds guter Lehre sowie die Identifikation bzw. Entwicklung valider und reliabler Erhebungsinstrumente in deutschlandweiter Zusammenarbeit scheint erstrebenswert und würde eine Weiterentwicklung der gültigen LOM-Lehre darstellen.

## Background

There are currently around 92,000 undergraduate medical students in Germany, dispersed among 37 medical schools. Teaching quality must meet high standards, both in terms of content [1] and the coverage of interprofessionalism and scientificity [2]. Owing to the purpose of national rankings, “teaching outcome” at individual medical schools is sometimes used as a surrogate marker of “teaching quality”. The second state examination only represents factual knowledge, and the aggregate exams provide but few clues to the specific strengths and weaknesses of individual curricula. Thus medical schools need other data sources to assess their teaching quality. One popular source are student evaluations of teaching. Depending on the survey instrument that is used, up to four dimensions of teaching quality can be mapped. According to Gibson et al., the structural and procedural characteristics of teaching as well as the didactic skills of the teachers and the student learning outcome are differentiated [3]. However, many evaluation tools focus on structural and process-related parameters [4] and only ask for student satisfaction with teaching. It is known that, particularly, global assessments by students are subject to a variety of confounding factors (e.g., individual characteristics such as gender, interest in subject, level of performance) [5]. Such a bias of the evaluation is problematic because at some medical schools the results are discussed as the basis of a performance-based allocation of resources (*Leistungsorientierte Mittelvergabe*, *LOM*) for teaching (teaching-LOM). In research, a defined benefit allocation has become well established. Although the parameters and algorithms used in research evaluation are criticized heavily [6], there has been a mismatch between incentives for good research and those for good teaching. Although a large amount of money flows into the basic equipment for teaching, the quality of teaching is often not sufficiently considered in resource allocation. Therefore, the perception of many scientists is that commitment to teaching pays off less than involvement in research.

So far, there is a lack of comprehensive data for German medical schools regarding their evaluation practices and the design of LOM algorithms in teaching. Against this background, the Working Group on Evaluation of Performance in Medical Research and Teaching of the Association of Scientific Medical Societies in Germany (AWMF) and the Medical School Association (MFT) have set the common goal of analyzing current evaluation practices at medical schools in Germany. This paper shows the results of a survey conducted at these schools.

## Methods

A standardized questionnaire was developed in a multi-stage procedure and piloted.

The questionnaire covered the following aspects of teaching evaluation and LOM awarding practice in eleven parts:

1. grounding of evaluation,
2. objects of evaluation,
3. persons involved in the evaluation,
4. regularity and frequency of evaluation of the curriculum,
5. format of evaluation by students,
6. format of the evaluation by academic teachers,
7. content of the evaluation by students,
8. use of objective data for quality assurance and evaluation,
9. processing and distribution of the evaluation results,
10. consequences of the evaluation,
11. allocation of funds for teaching.

The questionnaire mainly included yes/no questions, sometimes with the possibility of supplementing free text information. Occasionally, numbers were requested, especially for the range of the evaluations, the response rates of student evaluations, and the allocation of funds by the faculties.

The questionnaire was sent in July 2013 via the office of the Medical School Association to all medical schools in Germany. Medical schools were asked for written answers from the responsible staff and for sending relevant materials. In case of non-response, a reminder was issued at the end of August, further missing data were requested by phone in November and December. The (German) questionnaire is available from the authors upon request.

## Results

30 German medical schools participated in the survey with 33 study programs (23 standard curricula, 6 model curricula, 3 reformed standard curricula, and 1 degree program in molecular medicine) (response rate 83%).

The results are presented below according to the above-mentioned sections of the questionnaire.

### Grounding of evaluation

Only 21% of all study programs had a dedicated evaluation system. The majority relied on evaluation regulations of the respective university or on corresponding decisions of the departmental council. The majority (85%) cited their evaluation practice as “grown by experience”, and 75% think that it is scientifically justified. Regarding the data source, 40% referred to external and 36% to internal survey instruments. In addition, points such as relevant knowledge in methodology research or evaluation standards as defined by various groups (e.g., the German Evaluation Society (*DeGEVal*), the working group on evaluation, and the training committee) were mentioned. 

### Objects of the evaluation and involved groups of people

Most frequently, subjects (70%), individual courses (67%), and study sections (64%) are evaluated. In particular, the internship (PJ) is relevant to evaluation for 85% of the programs. Also, teachers are evaluated in three out of four medical schools. About half of the medical schools evaluate examinations (quality and results) and graduates. All degree programs use student evaluations, 45% involved teachers and 20–25% internal panels, but also external reviewers.

### Regularity and frequency of evaluation in the core curriculum

In less than 50% of the study programs, a comprehensive evaluation of all types of events and groups of persons takes place. Only courses in special medical disciplines or cross-sectional areas are consistently evaluated by about two thirds of medical schools; individual segments of the curriculum (e.g., preclinical, clinical, practical internship) in 42%; and teachers (27% to 36%) as well as examinations (33%) in about one third.

### Format of evaluations by students and academic teachers

Nearly all study programs (97%) use online formats for student evaluations, and about 50% (additionally) utilize paper-based formats for evaluations. The majority (94%) are evaluated outside of course times, mainly before or after the final exam. However during ongoing courses, evaluations take place in about 46%. The number of questions in the questionnaire varies greatly (1 to 140 items). Ordinal or interval-scaled items (e.g., grades, percentages) and free-text comments are most commonly used, as well as dichotomous, open and multiple choice questions. 

The reported response rates of student evaluations average approximately 60%. Incentives for increased returns, such as transparency (18%) and student bonus schemes (33%), as well as coercion and negative consequences, are only sporadically described as helpful. Other forms of evaluation include debriefing (54%) and reports from semester spokespersons (48%). Interviews (15%) and focus groups (21%) are less frequently used.

In the course evaluation by academic teachers, the majority (80%) choose a structured approach.

In 40% of the cases all teachers are evaluated; in 20% a selection of teachers are evaluated by committees.

### Content of the evaluation by students

Using student evaluations, structural and process parameters as well as the overall impression of the teaching is captured. The subjective relevance of the educational content for examinations and practice, the content structure and overall satisfaction with educational events or overall grades are most frequently rated by students (>80%). For further details see Table 1 (Tab. 1).

### Use of objective data for quality assurance and evaluation

94% of medical schools use objective data for internal or external quality assurance of teaching. Particularly frequently (>80%) the second state examination (pass rate in the reference cohort, failure rate) was named, followed by average study duration, average score in the second state examination and number of graduates (79% each), passing of the first state examination, and further qualifications of the teachers (76% each).

Ratio of support, number and quality of doctorates, research projects or publications on teaching, as well as average scores and subject-specific evaluations in the first state examination are less relevant parameters for quality assurance (50–70%).

### Processing and distribution of the evaluation results

The results of the evaluation are mostly written as reports and regularly stored electronically in a protected area. Usually, deans’ offices (79%) and professors responsible for issuing certificates (76%) are actively informed about the results, often also teaching coordinators (67%), individual academic teachers (61%), and students (64%). An active communication of results takes place in more than 40%; and in almost 10% of all cases, the (external) public is actively informed.

### Consequences of the evaluation

In two out of three faculties, teaching quality is rated according to fixed criteria/categories; in 82%, courses and academic teachers are also assessed in relation to each other. Mostly (82%) there are feedback talks with academic teachers, less frequently feedback on teaching content in cross-sectional areas (73%), medical subjects (61%), and modules (55%). In addition, just under 80% (79%) of the degree programs have provided consequences for particularly positive but also particularly negative evaluation results. For positive results, the main reported consequence (54%) is reward by teaching-LOM. Only occasionally, awards for the best lecturer or a bonus for promotions were mentioned. In contrast, 73% of study programs provide training and support for teachers with particularly negative outcomes, followed by face-to-face interviews (45%) and negative effects on teaching-LOM (21%).

### Allocation of funds for teaching 

**Figure 1:**
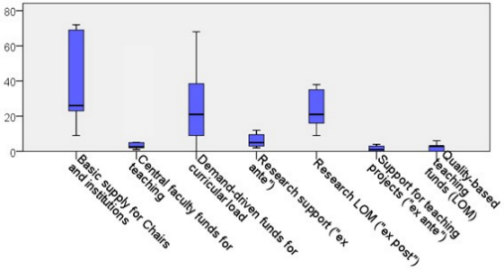
Relative distribution of state funds at medical school

Seventeen medical schools answered the questions about their internal distribution of resources. These data are shown in Figure 1 (Fig. 1). It becomes clear here that the three most important items represent the general basic supply of the Chairs as well as the curricular basic equipment and the evaluation-based research LOM. In contrast, evaluation-based funds for teaching play a minor role, as well as application-based research and teaching support. With an average of 1.6% (minimum 0%, median 3%, maximum 6%), the evaluation-based teaching-LOM tends to account for a small part of the state funds.

In 60%, the teaching-LOM follows a fixed algorithm. With just under 70%, it benefits clinics and institutes in particular, much less complete modules (15%) or individuals (21%).

## Discussion

The results of the survey are in agreement with the current literature [4], in so far as evaluation instruments used in Germany primarily assess structural and procedural aspects as well as students’ overall impression of teaching quality. Teaching outcome – mostly defined as students’ learning success – is either assessed by student ratings of their own perceived learning outcome, or it is inferred from student performance in high-stakes examinations. A systematic evaluation of teachers is rare.

For the dimensions “process” and “teacher”, the literature review by Schiekirka et al. [4] already identified numerous survey instruments for medical education with good to very good reliability. About one third of faculties use such instruments or use their scientific base for the development of own instruments. However, it is unclear whether the used instruments are equivalent to those already identified. Furthermore, it is uncertain how the instruments were developed, whether they were psychometrically tested and to what extent they meet established quality criteria. In 88% of medical schools, global grades are used to assess teaching quality. Although these evaluations provide a rough idea of student satisfaction with teaching, they are not considered valid measures of teaching quality due to the strong bias introduced by various construct-irrelevant confounders [5]. Data show for example that students with a high initial interest in a course generally tend to rate it more positively than those with a low interest [7], [8]. Further studies found a positive correlation between exam performance and student ratings for an anatomy course [9], [10]. In this context, the importance of a clear definition of the construct of good teaching underlying the evaluation should be emphasized: Only after it has been clearly defined what is meant by "good teaching" can one identify and use an instrument that measures precisely this construct. Conversely, the interpretation of existing evaluation data should only refer to that particular construct (e.g., structural conditions) and should not be generalized to other aspects of the quality of teaching (e.g., didactic skills of academic teachers). 

However, the psychometric examination of the evaluation tools used, minimization of distorting effects, as well as the coverage of all four dimensions suggested by Gibson [3] are urgently needed in order to be able to validly assess and optimize teaching quality.

Even when the result of teaching is inferred from seemingly objective exam data, some difficulties need to be kept in mind. In order to provide valid data, examinations need to be aligned to learning objectives and instructional methods [11] and thereby free of construct-irrelevant variance [12], [13]. They must also meet international quality standards [14], which cannot be taken for granted [15]. 

These requirements become even more important when the distribution of teaching-LOM is based on evaluation results and examinations. So far, the financial remuneration of teaching has mainly been part of the basic equipment of Chairs or has been based on teaching quantity. Quality-based funding for teaching, unlike research, plays only a minor role. Also other gratifications for high quality teaching are only occasionally given. 

Due to the current heterogeneity of the evaluations, it is not possible or at least of limited value to compare the results of teaching evaluations across medical schools. Although a certain degree of comparability can be established by comparing students’ results in central written exams, the validity of the data must be discussed against the background of the construct of good teaching chosen in each case. In terms of rewarding good teaching, initial data show that teachers are currently more motivated by student feedback, but they also have a positive attitude towards financial incentives [16], [17]. Thus, it seems to be unrewarding to exclusively emphasize financial incentives; instead, other aspects of appreciation and specific support (for example by improving the organizational conditions for teaching and career opportunities) can help to increase and sustain motivation of academic teachers [18]. Just as good research performance leads to both immaterial or career-related and direct material appreciation, an incentive system for good teaching should be based on a broad range of rewards in order to overcome the existing imbalance.

## Conclusions

This first systematic survey of German medical schools on the practices used for evaluating medical teaching has shown that the evaluation instruments used have similar content and methodology, and above all record structural and procedural aspects as well as students’ overall impression of teaching. Yet, there is considerable heterogeneity regarding the instruments actually used. A nationwide consensus on a general construct of good medical teaching as well as the identification or development of valid and reliable evaluation instruments in a nation-wide cooperation appears sensible.

## Notes

### Competing interests

The authors declare that they have no competing interests.

### Authorship

The authors Raupach T and Herrmann-Lingen C contributed equally to this work.

## Acknowledgements

We thank the AWMF for financial support, the MFT office (Dr. Corinne Dölling) for logistical support, Mrs. Sabine Gluth for the completion and reporting of the survey data, the staff of the Deans of Studies for answering the questionnaires, and the members of the AWMF Committee for Performance Evaluation in Research and Teaching and the MFT Working Group on Teaching for their support in implementation and interpretation of the survey.

## Figures and Tables

**Table 1:**
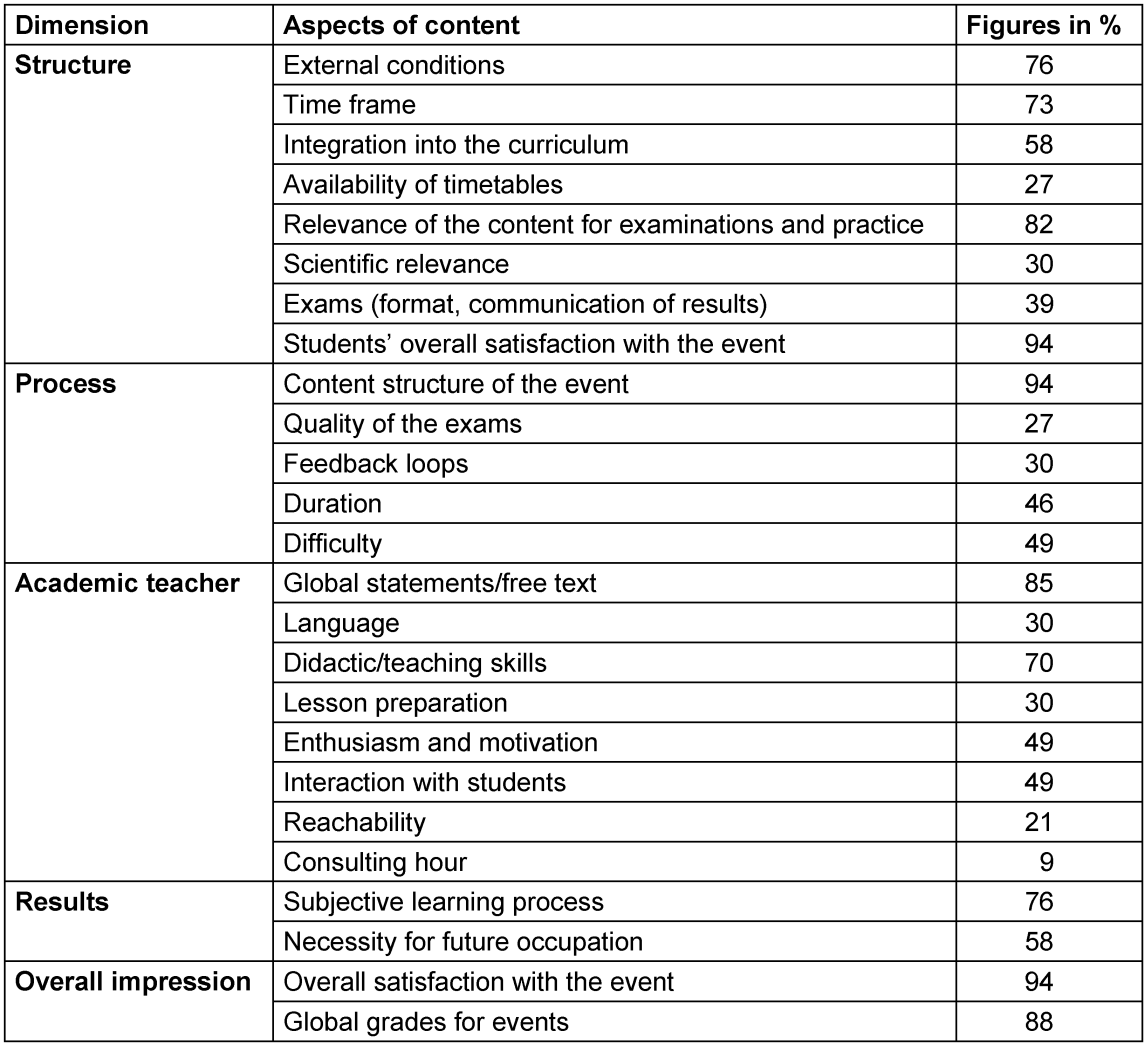
Aspects that are captured by educational event evaluations by students
